# HDAC inhibition as a treatment concept to combat temsirolimus-resistant bladder cancer cells

**DOI:** 10.18632/oncotarget.22454

**Published:** 2017-11-06

**Authors:** Eva Juengel, Ramin Najafi, Jochen Rutz, Sebastian Maxeiner, Jasmina Makarevic, Frederik Roos, Igor Tsaur, Axel Haferkamp, Roman A. Blaheta

**Affiliations:** ^1^ Department of Urology, Goethe-University, Frankfurt am Main, Germany; ^2^ Current address: Department of Urology and Pediatric Urology, Mainz University Medical Center, Mainz, Germany

**Keywords:** bladder cancer, temsirolimus-resistance, HDAC inhibition, valproic acid (VPA), tumor growth

## Abstract

**Introduction:**

Although the mechanistic target of rapamycin (mTOR) might be a promising molecular target to treat advanced bladder cancer, resistance develops under chronic exposure to an mTOR inhibitor (everolimus, temsirolimus). Based on earlier studies, we proposed that histone deacetylase (HDAC) blockade might circumvent resistance and investigated whether HDAC inhibition has an impact on growth of bladder cancer cells with acquired resistance towards temsirolimus.

**Results:**

The HDAC inhibitor valproic acid (VPA) significantly inhibited growth, proliferation and caused G0/G1 phase arrest in RT112^res^ and UMUC-3^res^. cdk1, cyclin B, cdk2, cyclin A and Skp1 p19 were down-regulated, p27 was elevated. Akt-mTOR signaling was deactivated, whereas acetylation of histone H3 and H4 in RT112^res^ and UMUC-3^res^ increased in the presence of VPA. Knocking down cdk2 or cyclin A resulted in a significant growth blockade of RT112^res^ and UMUC-3^res^.

**Materials And Methods:**

Parental (par) and resistant (res) RT112 and UMUC-3 cells were exposed to the HDAC inhibitor VPA. Tumor cell growth, proliferation, cell cycling and expression of cell cycle regulating proteins were then evaluated. siRNA blockade was used to investigate the functional impact of the proteins.

**Conclusions:**

HDAC inhibition induced a strong response of temsirolimus-resistant bladder cancer cells. Therefore, the temsirolimus-VPA-combination might be an innovative strategy for bladder cancer treatment.

## INTRODUCTION

Bladder cancer is the fourth most common cancer diagnosed in men [1]. In 2016, an estimated 76,960 new patients will be diagnosed with bladder cancer in the US [2] and about 430.000 cases worldwide [3], 16,390 will probably die from complications of this disease in the US [2] and about 160.000 worldwide [3]. Around thirty percent of the cases are already diagnosed as muscle-invasive urothelial carcinoma and most of them have locally advanced or disseminated disease at diagnosis [4]. The standard treatment for patients with muscle-invasive bladder cancer (MIBC) includes neoadjuvant cisplatin-based chemotherapy along with radical cystectomy. Although these regimens have a high response rate, they are generally non-curative, with median progression-free survival of approximately 8 months and a 5-year overall survival rate of 5–10% [1, 3]. New therapeutic approaches are urgently needed and research is focused on development of targeted therapies, which may be more effective [5] than the current protocol. Next generation sequencing of invasive urothelial carcinoma has identified the phosphatidylinositol 3-kinase/protein kinase B/mechanistic target of rapamycin (PI3K/Akt/mTOR) pathway as a potential therapeutic target [6]. mTOR pathway activation has been shown to be involved in urothelial bladder cancer tumorigenesis and to be a predictor of disease progression and cancer specific survival [7, 8]. Data of the Cancer Genome Atlas (TCGA) confirmed these findings [9]. mTOR inhibitors have been evaluated as anticancer agents, some of which are already approved for the treatment of metastatic renal cell carcinoma (temsirolimus, everolimus), mantle cell lymphoma (temsirolimus), breast cancer (everolimus) and pancreatic neuroendocrine tumors (everolimus) [10]. Disappointingly, a phase II study with everolimus, given as a single agent in bladder cancer, did not show the efficacy that might have been expected [11]. In fact, mTOR inhibition revealed heterogeneous responses, indicating anti-tumor effects in some cases, while others exhibit intrinsic or acquired resistance to the drug both in preclinical or clinical settings [10]. Mechanisms underlying resistance are various and include loss of mTOR inhibition, feedback activation of PI3K and Akt [12].

A combination with other compounds might be promising. Based on earlier studies, we postulate that modulation of the histone acetylation status by a histone deacetylase (HDAC) inhibitor might be an attractive strategy to improve an mTOR inhibitior-based regime. Notably, HDAC suppression may not only elevate the therapeutic efficacy of an mTOR inhibitor per se [13] but may overcome resistance towards this respective class of drugs [14–16]. HDAC inhibitors have been identified to restore epithelial differentiation and to abrogate growth in different cancer cells, including bladder cancer cells [17]. Several data point to the principal importance of a combined HDAC-mTOR inhibitor-based regime to optimize tumor treatment. A single molecule inhibitor targeting both HDAC activity and PI3K signaling has recently been developed, which induced greater tumor growth inhibition and pro-apoptotic activity than single-target PI3K or HDAC inhibitors *in vitro* and *in vivo* [18]. Accordingly, combining the HDAC inhibitor vorinostat with the mTOR inhibitor MLN0128 increased the expression of pro-death genes and the sensitivity to apoptotic triggers [19]. In trametinib/dabrafenib-resistant melanoma cells, addition of the HDAC inhibitor AR42 with pazopanib contributed to significantly reduced tumor growth *in vitro* and *in vivo* [20].

Since the relevance of HDAC suppression for drug-resistant bladder cancer cells has not yet been evaluated, we explored whether the HDAC inhibitor valproic acid (VPA) exerts anti-tumor properties on a panel of temsirolimus-resistant bladder cancer cell lines.

## RESULTS

### HDAC inhibition causes growth and proliferation blockade of both temsirolimus sensitive and resistant cells

**Figure 1:**
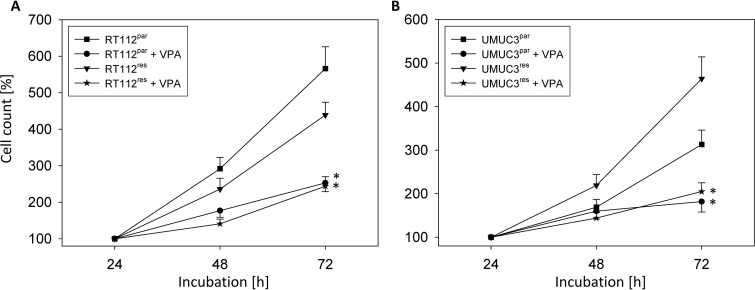
Growth of parental (par) and temsirolimus-resistant (res) bladder cancer cells, RT112 (**A**) and UMUC-3 (**B**). Temsirolimus-resistant cells were exposed to 1 μmol/ml temsirolimus three times a week. Cells were treated with VPA [1 mmol/ml] in the 96-well-plates for 24 h, 48 h and 72 h. Controls remained untreated. Cell number was set to 100% after 24h incubation. Bars indicate standard deviation (SD). ^*^indicates significant difference to untreated control cells, *p* ≤ 0.05. *n* = 5.

Cell growth of RT112^res^ was only slightly reduced when compared to RT112^par^ cells (Figure 1A), whereas growth of UMUC-3^res^ cells was even enhanced when compared to the respective parental control (Figure 1B). Incubation with VPA [1 mmol/ml] induced a significant growth inhibition of both RT112^par^ and RT112^res^ cells compared to the untreated cell sublines (Figure 1A). Growth suppression was also evoked when VPA was added to UMUC-3^par^ or UMUC-3^res^ cell cultures (Figure 1B).

**Figure 2:**
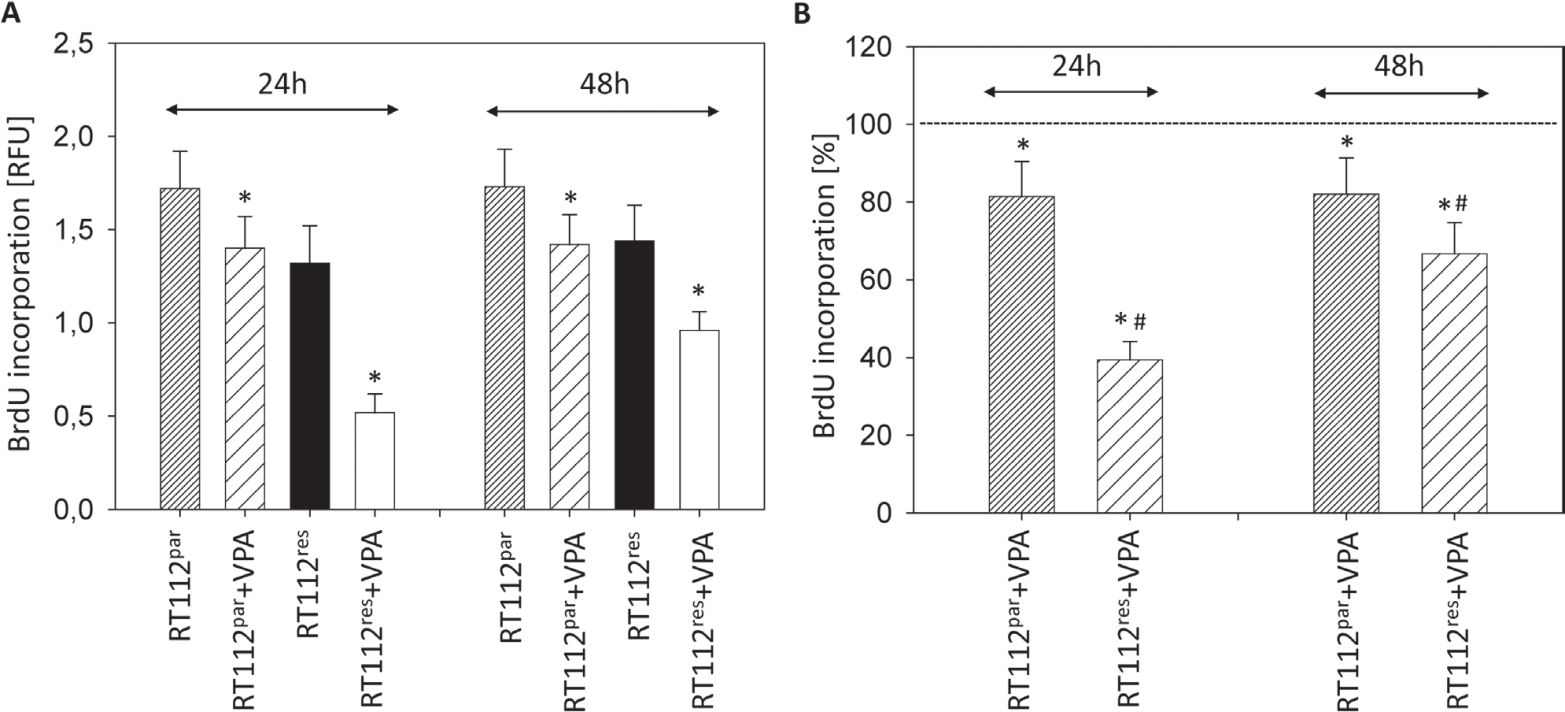
Proliferation of RT112^par^ and RT112^res^ Temsirolimus-resistant cells were exposed to temsirolimus [1 μmol/ml] three times a week. Tumor cells were further treated with VPA [1 mmol/ml] in the BrdU assay for 24 h or 48 h. Controls remained untreated. (**A**) BrdU incorporation [RFU] for each sample. (**B**) % difference of VPA treated cells to controls without VPA. Bars indicate standard deviation (SD). ^*^indicates significant difference to control, ^#^indicates significant difference to parental cells, *p* ≤ 0.05. *n* = 5.

**Figure 3:**
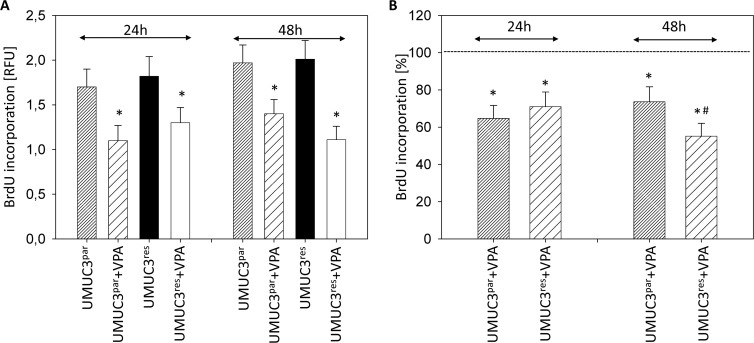
Proliferation of UMUC-3^par^ and UMUC-3^res^ Temsirolimus-resistant cells were exposed to 1 μmol/ml temsirolimus three times a week. Tumor cells were further treated with VPA [1 mmol/ml] in the BrdU assay for 24 h or 48 h. Controls remained untreated. (**A**) BrdU incorporation [RFU] for each sample. (**B**) % difference of VPA treated cells to controls without VPA. Bars indicate standard deviation (SD). ^*^indicates significant difference to control, ^#^indicates significant difference to parental cells, *p* ≤ 0.05. *n* = 5.

Evaluation of tumor cell proliferation revealed distinct tumor suppressive properties of VPA exerted on RT112^par^ and RT112^res^ cells (Figure 2A) and on UMUC-3^par^ and UMUC-3^res^ cells (Figure 3A). Interestingly, stronger effects of VPA were induced on the resistant cell cultures after 24 h (RT112) and 48 h (RT112 and UMUC-3) compared to the sensitive ones. Mean percentage of RT112 proliferation blockade was calculated to 18.6% versus 60.6% (24 h values, sensitive versus resistant) and 18.0% versus 33.3% (48 h values, sensitive versus resistant; Figure 2B). Mean percentage of UMUC-3 proliferation blockade was 26.3% versus 44.8% (48 h values, sensitive versus resistant; Figure 3B). Differences in the inhibitory efficacy of VPA on UMUC-3^par^ versus UMUC-3^res^ were not seen after 24 h. No significant apoptotic or necrotic activity of VPA has been detected, indicating that reduced cell growth and proliferation was not caused by apoptotic events (data not shown).

### HDAC inhibition results in G0/G1 cell cycle arrest

**Figure 4:**
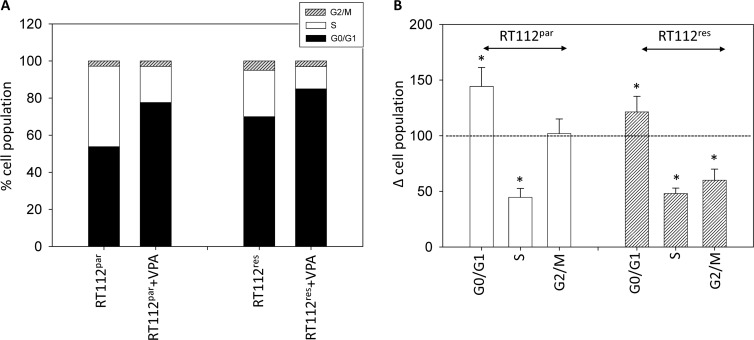
Cell distribution in the different cell cycle phases (**A**) Percentage of parental and resistant RT112 in G01/1, S and G2/M phase is indicated. Bladder cancer cells were pre-treated with VPA [1 mmol/ml] for 3 days. Controls remained untreated. One representative of three separate experiments is shown. (**B**) % difference of RT112^par^ and RT112^res^ exposed to VPA [1 mmol/ml] compared with the corresponding untreated controls. Control phases were set to 100%. Bars indicate standard deviation (SD). ^*^indicates significant difference to control, *p* ≤ 0.05. *n* = 5.

**Figure 5:**
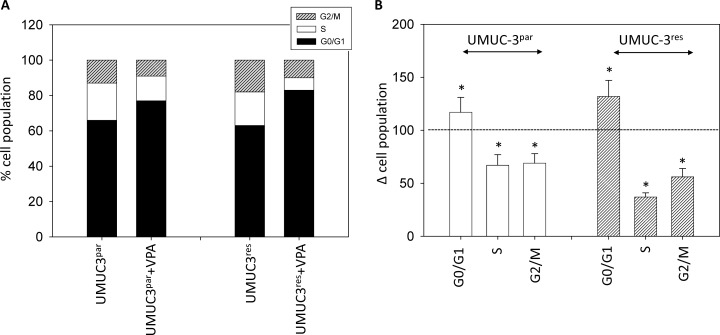
Cell distribution in the different cell cycle phases (**A**) Percentage of parental and resistant UMUC-3 in G01/1, S and G2/M phase is indicated. Bladder cancer cells were pre-treated with VPA [1 mmol/ml] for 3 days. Controls remained untreated. One representative of three separate experiments is shown. (**B**) % difference of UMUC-3^par^ and UMUC-3^res^ exposed to VPA [1 mmol/ml] compared with the corresponding untreated controls. Control phases were set to 100%. Bars indicate standard deviation (SD). ^*^indicates significant difference to control, *p* ≤ 0.05. *n* = 5.

The number of temsirolimus-resistant RT112 and UMUC-3 cells in G2/M increased, accompanied by a decrease in the number of S-phase cells (each compared to the respective drug sensitive control, Figures 4, 5). In addition, more RT112^res^ cells were recorded in G0/G1 (versus RT112^par^), whereas no differences were seen in the number of UMUC-3^par^ versus UMUC-3^res^ cells in this matter (Figure 4, Figure 5). VPA induced distinct accumulation of RT112^par^ in G0/G1 (144.2 +/− 17.2%) with a simultaneous decrease of S-phase cells (44.7 +/− 8.1%; Figure 4). The number of RT112^par^ in G2/M was not altered by VPA. With respect to RT112^res^ cells, VPA elevated the amount of G0/G1 phase cells (121.4 +/− 14.4%) and reduced both the amount of S-phase (48.0 +/− 5.2%) and G2/M-phase cells (60.7 +/− 10.0%; Figure 4).

Both, UMUC-3^par^ and UMUC-3^res^ cells were similarly modified by VPA, evidenced by a decrease of G2/M cells (parental cells: 69.3 +/− 9.7%; resistant cells: 56.8 +/ 8.4%), reduction of cells in the S-phase (parental cells: 67.5 +/− 10.2%; resistant cells: 37.0 +/ 4.1%), associated with an increase of both UMUC-3^par^ and UMUC-3^res^ in G0/G1 ((parental cells: 117.7 +/− 14.3%; resistant cells: 132.8 +/ 15.6%; Figure 5).

### VPA causes distinct modulations of cell cycle regulating proteins

**Figure 6:**
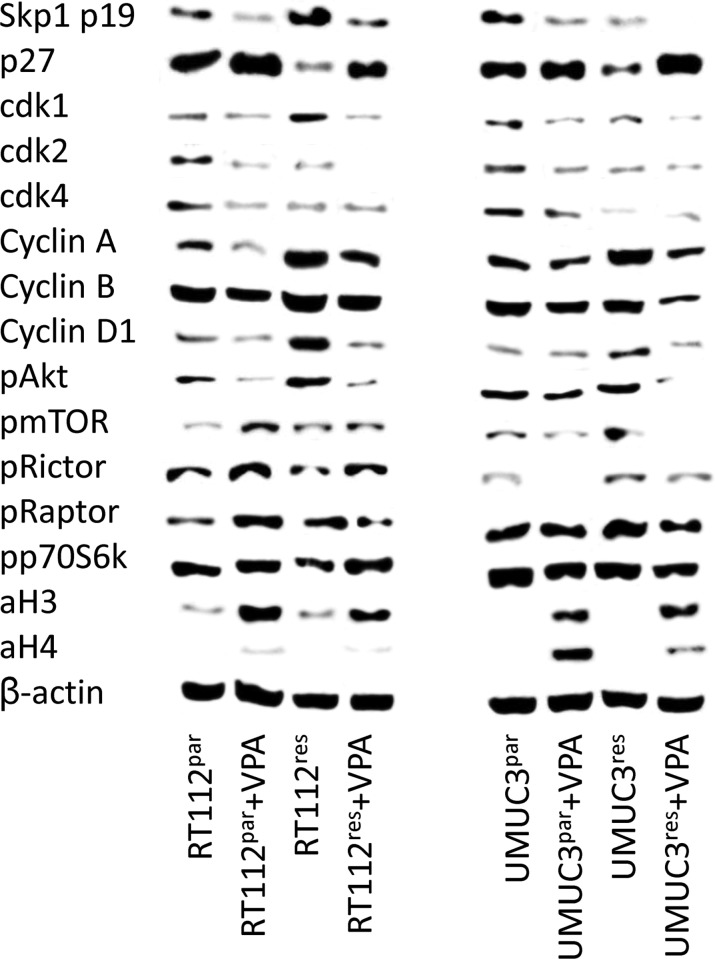
Protein expression profile of cell cycle regulating and targeted proteins in parental and temsirolimus-resistant RT112 (left) and UMUC-3 (right) cells after 3 days exposure to VPA [1 mmol/ml] and untreated controls ß-actin served as internal control. One representative of three separate experiments is shown.

Functional alterations in growth, proliferation and cell cycle progression induced by VPA were associated with distinct modulation of cell cycle regulating protein expression and activity (Figure 6). Concerning the protein expression pattern in RT112^res^ versus RT112^par^ cells, the following proteins were found to be up-regulated in RT112^res^ (Figure 6, left): Skp1 p19, cdk1, cyclin A, B and D1, pAkt, pmTOR and pRaptor. Diminished expression level in RT112^res^ versus RT112^par^ cells was related to p27, cdk2, cdk4, pRictor and pp70S6k. The acetylation status of histone H3 (aH3) did not change during resistance development (Figure 6, left).

VPA similarly acted on RT112^res^ versus RT112^par^ cells with respect to Skp1 p19, cdk1, cdk2, cyclin A, B and D1 and pAkt (all: protein suppression) and p27, pRictor and aH3 (all: protein elevation, Figure 6, left). Acetylation of histone H4 (aH4) was not detectable in both RT112^res^ and RT112^par^ cells; however, a very slight band appeared when cells were exposed to VPA. Differences between both cell sublines following VPA application have also been recorded. Cdk4 was diminished by VPA in RT112^par^ cells exclusively; pRaptor was enhanced in RT112^par^ but reduced in RT112^res^ cells. Phosphorylation of p70S6k (pp70S6k) became elevated in RT112^res^ cells but remained unchanged in RT112^par^.

In accordance with the RT112 data, UMUC-3^res^ have been characterized by an up-regulation of cyclin A, cyclin D1, pmTOR and pRaptor, and a down-regulation of p27, cdk4 and pp70S6k, each compared to UMUC-3^par^ cells (Figure 6, right). In contrast to the RT112 data, Skp1 p19 (distinctly), cdk1, cdk2 (both moderately) were suppressed in UMUC-3^res^, pRictor increased and cyclin B and pAkt remained unchanged. Acetylation of histones H3 and H4 (aH3, aH4) was not detectable in UMUC-3^res^ and UMUC-3^par^ cells.

In presence of VPA, Skp1 p19, cdk1, cdk2, cyclin A, cyclin B, pAkt, pmTOR and pRictor were all diminished in both UMUC-3^res^ and UMUC-3^par^ cells (Figure 6, right). Independent of the UMUC-3 cell subline, VPA additionally induced an up-regulation of p27 and strong expression of aH3 and aH4 (UMUC-3^par^ > UMUC-3^res^). Different effects of VPA on resistant versus sensitive tumor cells have also been observed. cdk4 increased in UMUC-3^par^ but decreased in UMUC-3^res,^ whereas cyclin D1 and pRaptor were not modified in UMUC-3^par^ but reduced in UMUC-3^res^.

### Decrease of cdk2 and cyclin A is involved in VPA-induced growth inhibition

**Figure 7:**
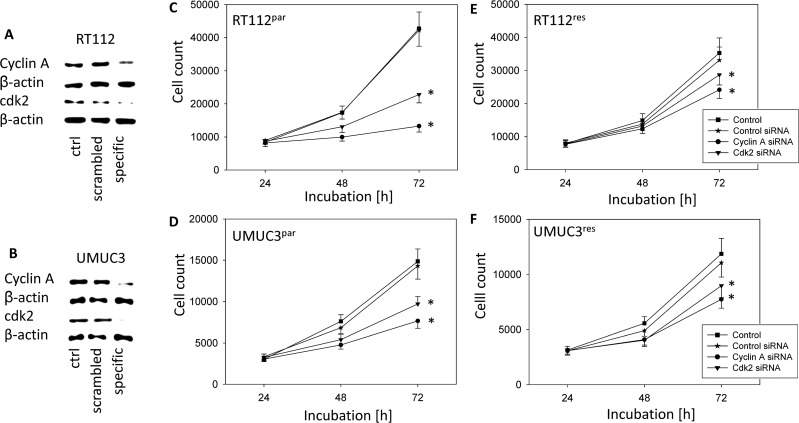
Functional blocking with siRNA targeting cdk2 and cyclin A of RT112 (upper panel) and UMUC-3 (lower panel) All Stars Negative Control siRNA served as transfection control (mock). Controls remained untreated. (**A**) and (**B**) Protein expression profile of cell cycle regulating proteins of RT112 and UMUC-3 cells after functional blocking with siRNA targeting cdk2 and cyclin A. ß-actin served as internal control. One representative of three separate experiments is shown. (**C**–**F**) Tumor cell growth of blocked bladder cancer cells, (C) RT112^par^, (D) UMUC-3^par^, (E) RT112^res^ and (F) UMUC-3^res^. Bars indicate standard deviation (SD). ^*^indicates significant difference to control, *p* ≤ 0.05. *n* = 5.

Common to all tumor cells analysed, cdk2 and cyclin A were distinctly suppressed by VPA. Therefore we evaluated the particular role of the cdk2-cyclin A axis in tumor growth control by blocking their function using siRNA. Knock-down of cdk2 and cyclin A resulted in significant cell growth inhibition in all tumor sublines, compared to the untreated cells and the mock control (Figure 7C–7F). Down-regulation of cyclin A had a stronger effect than blocking cdk2. Knock-down efficacy of cdk2 and cyclin A protein expression was verified by Western blot analysis (Figure 7A, 7B).

## DISCUSSION

To our knowledge, this is the first manuscript dealing with temsirolimus-driven resistance mechanism in bladder cancer and the potential of VPA in combating resistance. Common to both resistant cell lines, RT112^res^ and UMUC-3^res^, cyclin A, cyclin D1, pmTOR and pRaptor were up-regulated during chronic drug treatment with temsirolimus. Concerning the mTOR molecule, two functionally distinct sub-structures exist: mTOR complex 1 (mTORC1), which (among others) contains the Rapamycin-sensitive adapter protein of mTOR (Raptor), and mTOR complex 2 (mTORC2), which includes the Rapamycin-insensitive companion of mTOR (Rictor). The mechanistic details of mTORC1-mTORC2 crosstalk are not completely understood. Evidence has been provided confirming Raptor (mTORC1) as the main driving force for mitosis induction and progression and, inversely, resistance induction caused by chronic mTOR blockade has been associated with increased Raptor activation [14]. Since cyclin D1 is under the control of mTORC1 [21, 22], it might not be surprising to see an up-regulation of cyclin D1 in the context of pRaptor increase. A positive correlation between cyclin A and mTOR expression has also been shown [15, 23], although it is not yet clear whether cyclin A might be regulated by mTORC1, mTORC2 or by both.

In contrast to the behavior of cyclin A, cyclin D1, pmTOR and pRaptor, p27 was massively reduced in the temsirolimus-resistant tumor cell lines. Clinical studies on bladder cancer patients point to a negative correlation between p27 expression and recurrence-free survival, as well as between p27 expression and overall survival in this matter [24–26]. Based on a bladder cancer cell model, expression of p27 seems to be directly mediated through an mTOR-dependent mechanism [27], presumably via mTORC1 [28]. Therefore we assume that long-term administration of temsirolimus to bladder cancer cells may result in a feedback mechanism characterized by re-activation of Raptor, associated with cyclin A and cyclin D1 elevation and loss of p27. This scenario may accelerate mitotic cycling and tumor progression. However, apart from similarities among the cell lines, cell line specific alterations have also been noted. E.g. RT112^res^ revealed enhanced Skp1 p19 and cdk1 and reduced pRictor, whereas the opposite was true for UMUC-3^res^ cells. Bearing in mind that cell growth of RT112 versus UMUC-3 cells was differentially influenced by chronic temsirolimus treatment (RT112^res^ < RT112^par^ and UMUC-3^res^ > UMUC-3^par^), a different molecule expression pattern might reflect different drug sensitivity. However, this assumption is speculative and requires further evaluation.

HDAC suppression by VPA contributed to a significant reduction of bladder cancer cell growth and proliferation, not only of the parental but, most importantly, of those sublines with acquired resistance towards temsirolimus. Due to this, we conclude that HDAC inhibition might be an innovative strategy to overcome mTOR-driven resistance processes. The principal significance of histone modifications on bladder cancer progression has already been well documented. HDAC inhibition mediates apoptosis [29], delays cell cycle progression [30] and blocks adhesive events of bladder cancer cells [31]. Novel data revealed an increased transcription of DNA repair genes by VPA [32]. There is also evidence that targeting HDAC might reverse resistance towards a considerable panel of chemotherapeutic drugs such as cisplatin [33], methotrexate [34], paclitaxel [35], gemcitabine [36], temozolomide [37], gefitinib [38] and epirubicin [39]. Since blocking of HDAC also counteracts resistance to tyrosine kinase inhibitors [40], epigenetic repression might be hypothesized as being an effective strategy to optimize current anti-tumor protocols [41–43].

In good accordance with our results, combined HDAC-mTOR blockade delayed the time to resistance towards the mTOR inhibitor ridaforolimus in a clinical renal cancer study [44] and *in vitro* studies showed a distinct impact of HDAC suppression on growth and proliferation of tumor cells with acquired resistance to the mTOR inhibitor everolimus [14, 15]. The effect of VPA on bladder cancer progression was associated with an accumulation in the G0/G1-phase and concomitant decrease of S-phase cells. Reports point to a G0/G1-phase arrest, paralleled by a reduction of S-phase cells, as the main mechanism of VPA on several tumor entities such as oral squamous cell carcinoma [45], ovarian cancer [46], endometrial cancer [47], renal cell [15] and prostate carcinoma cells [48]. Interestingly, G2/M phase cells were also diminished in both UMUC-3^par^ and UMUC-3^res^ cultures, whereas VPA lowered the number of RT112^res^ but not of RT112^par^ G2/M phase cells. The different response of the RT112 cell sublines to VPA (in terms of G2/M counts) can be interpreted in two ways. Either this phenomenon reflects a different mode of action of VPA on the resistant versus sensitive cells, or an unspecific effect is seen here. In support of the first assumption, the BrdU incorporation assay demonstrated stronger effects of VPA on the resistant RT112 subline, particularly after 24 h incubation. However, only very few RT112^par^ cells have been counted in G2/M, making a further reduction under VPA unlikely (favoring the second assumption). Indeed, experiments on renal cell cancer cells showed a variable influence of VPA on G2/M cell populations which depended on the basal G2/M count [49]. Nevertheless, both assumptions are hypothetical and not underlined by fundamental data.

As a common mechanism, delay in cell cycle progression of RT112 and UMUC-3 cells caused by VPA was associated with up-regulation of p27 and acetylation of H3 and H4. Immunohistochemical evaluation of bladder cancer specimens revealed that p27 may serve as a prognostic biomarker as well as a promising therapeutic target [50, 51]. In fact, loss of p27 expression correlated with overall and recurrence-free survival [24, 52] and was associated with stage, grade, DNA ploidy and lymph-node involvement [53]. Recently, acetylation of histone H3 or H4 was demonstrated to be directly linked to the p27 promoter [54, 55]. Since aH3 and aH4 expression levels correlated with the p27 level in our experiments, we presume that epigenetic regulation of gene transcription by histone acetylation is (at least partially) responsible for increasing p27 and p27 driven cell growth control. Remarkably, the influence of VPA on p27 was strongest in the drug-resistant bladder cancer cell lines. p27 has been associated with G0/G1 S-phase transition and BrdU incorporation rate [56, 57], which might explain why BrdU uptake was diminished to a lesser extent by VPA in the temsirolimus sensitive cell lines compared to the resistant ones (RT112^res^−24 + 48 h; UMUC-3^res^-24 h) and why the S-phase was diminished to a greater extent in UMUC-3^res^ compared to UMUC-3^par^.

As a further common mechanism, VPA acted on the cyclin cdk axis by suppressing cdk 1 and 2 along with cyclin A and B. This is important as these molecules are crucially involved in cell growth regulation. Patient data have demonstrated a positive correlation between cyclin A-cdk2 level and metastatic progression of bladder cancer [58–60]. siRNA knockdown studies on UMUC-3 and RT112 cell lines revealed that VPA induced loss of cdk2 and cyclin A might be one prominent mechanism causing VPA to slow down mitotic activity. Former experiments on bladder cancer cell culture models showed that the cyclin B-cdk1 axis is also closely involved in tumor growth and proliferation, and that down-regulation of cyclin B/cdk1 causes a distinct delay in cell cycle progression [30, 61].

The mechanism of VPA on cdk-cyclin expression is of high clinical relevance. Aberrant activation of the cdk-cyclin family with subsequent proliferation through the deregulation of cell cycle control has been recognized as one of the key hallmarks of cancer [62]. Recent publications highlight the role of cdk members as potent targets for cancer therapeutics, with the hope of achieving cdk-cyclin inhibition in preventing the emergence of resistance to multiple targeted therapies across various cancer types [63]. In this regard, VPA may exert the resistance-preventing properties as desired by Whittacker and coworkers [63]. Importantly, VPA is well established in the treatment of epilepsy and relatively safe, with a low toxicity and convenient pharmacokinetic properties, which may recommend VPA as a useful adjuvant in the treatment of bladder cancer once resistance has been developed. Although reports are not available dealing with this issue, VPA increased the sensitivity of bladder cancer to mitomycin C, cisplatin and doxorubicin *in vitro* and *in vivo* [64], which is in accordance with our statement.

Nevertheless, there are also some discrepant data which need closer discussion. VPA reduced Akt-mTOR signaling in both drug-resistant and drug-sensitive UMUC-3 sublines. In contrast, mTOR and Raptor were activated by VPA in the drug-sensitive RT112^par^ cells, and pRictor was in fact enhanced in both RT112^par^ and RT112^res^. Data provided in the literature are also inconsistent. Suppression of the Akt/mTOR signal pathway in prostate cancer cell lines by VPA has been documented by Xia et al. [65], whereas others observed elevated Akt activation by VPA in the same culture system [13]. Based on *in vivo* rat models, VPA exposure decreased pmTOR in one experimental approach [66] but increased pmTOR in the other approach [67]. Two scenarios should be considered when interpreting the results. Activation of Akt-mTOR might point to resistance development towards VPA. In fact, we recently not only provided evidence of a diminished Akt content in VPA sensitive tumor-bearing animals, but also a massive accumulation of Akt in VPA non-responders [68]. Moreover, histone acetylation may activate Akt-mTOR signaling. Cross-communication has recently been observed in a prostate cancer cell line in a manner that enhanced histone H3 and H4 acetylation triggered elevated Akt-mTOR activity, particularly seen with pRictor [69]. The clinical relevance of this finding is not yet clear. VPA counteracted temsirolimus-driven resistance processes in two different bladder cancer cell lines. This property qualifies VPA as a highly valuable compound which may minimize the rapid onset of resistance induction caused by chronic suppression of mTOR. Since VPA may also (re)activate Akt-mTOR under certain circumstances, the question arises about the optimum treatment option. We have recently demonstrated that simultaneous targeting of both HDAC and mTOR delays the time to resistance towards the mTOR inhibitor [16]. With respect to these data, combined use of an HDAC and mTOR inhibitor might be superior to a regimen based on mTOR inhibition followed by HDAC blockade, once resistance towards the mTOR inhibitor has been developed. However, this assumption requires further evaluation. Preclinical evaluation of dual mTOR-HDAC inhibition in non-Hodgkin lymphoma cells showed that the mechanisms of effectiveness of both drugs were largely retained [70]. Patients with renal cell carcinoma experienced prolonged disease stabilization under co-administration of the mTOR inhibitor ridaforolimus and the HDAC inhibitor vorinostat in a phase I study [44]. In a similar protocol, simultaneous application of the mTOR inhibitor sirolimus and vorinostat led to stable disease in hepatocellular carcinoma patients [71].

In addition to impairing the tumor cell growth of the resistant cells, there is apparent evidence that the combined inhibition of HDAC and mTOR might also have an impact on the metastatic spread. This aspect is interesting, as advanced cancers and therapy resistance are accompanied by the occurrence of metastases. In non-small-cell lung cancer (NSCLC) combined HDAC and mTOR inhibition resulted in a synergistic decrease of migration and invasion *in vitro* and diminished metastasis rates *in vivo* [72]. Notably, the effect of HDAC blockade on the metastatic properties has also been demonstrated on osteosarcoma cells [73]. Whether treatment with an HDAC inhibitor may also modulate the metastatic potential of temsirolimus-resistant bladder cancer cells is as yet speculative. The results of ongoing studies in our group will shed light on this aspect. From our present data, we postulate that VPA might reverse bladder cancer cell therapy resistance to temsirolimus by at least partially blocking the cdk2/cyclin A axis. Further investigations should evaluate the effect of concomitant HDAC and mTOR inhibition in bladder cancer cells.

## MATERIALS AND METHODS

### Cell cultures and treatment

RT112 and UMUC-3 (ATCC/LGC Promochem GmbH, Wesel, Germany) bladder carcinoma cells were grown and cultured in RPMI 1640 supplemented with 10% fetal calf serum (FCS), 20 mmol HEPES buffer, 1% glutamax and 1% penicillin/streptomycin (all: Gibco/Invitrogen; Karlsruhe, Germany) in a humidified, 5% CO2 incubator. RT112 is an invasive (pathological stage T2) moderately differentiated (grade 2/3) model of human bladder cancer, UMUC-3 a high grade 3 invasive bladder cancer. In all experiments, treated to non-treated tumor cell cultures were compared. Resistance towards temsirolimus was induced by treating tumor cells with stepwise ascending concentrations from 1 nmol/ml up to 1 μmol/ml. The tumor cells were further exposed to 1 μmol/ml temsirolimus three times a week for over one year. Tumor cells, resistant to temsirolimus, were designated UMUC3^res^ and RT112^res^. The parental control cells are named UMUC3^par^ and RT112^par^. Valproic acid (VPA) (G. L. Pharma GmbH, Lannach, Austria) was applied at a final concentration of 1 mmol/ml to the cells for 1-3 days. Control cell cultures remained untreated. To examine toxic effects of amygdalin, cell viability was determined by trypan blue (Gibco/Invitrogen).

### Measurement of tumor cell growth, proliferation and apoptosis

Cell growth was assessed using the 3-(4,5-dimethylthiazol-2-yl)-2,5-diphenyltetrazolium bromide (MTT) dye reduction assay (Roche Diagnostics, Penzberg, Germany). Bladder cancer cells (50 μl, 1 × 10^5^ cells/ml) were seeded onto 96-well tissue culture plates. After 24, 48 and 72 h, 10 μl MTT (0.5 mg/ml) were added for an additional 4 h. Thereafter, cells were lysed in a buffer containing 10% SDS in 0.01 M HCl. The plates were incubated overnight at 37°C, 5% CO2. Absorbance at 550 nm was determined for each well using a microplate enzyme-linked immunosorbent assay (ELISA) reader. After subtracting background absorbance, results were expressed as mean cell number.

Cell proliferation was measured using a BrdU cell proliferation enzyme-linked immunosorbent assay (ELISA) kit (Calbiochem/Merck Biosciences, Darmstadt, Germany). Tumor cells (50 μl, 1 × 10^5^ cells/ml), seeded onto 96-well plates, were incubated with 20 μl BrdU-labeling solution per well for 8 h, fixed and detected using anti-BrdU mAb according to the manufacturer's instructions. Absorbance was measured at 450 nm using a microplate ELISA reader.

To evaluate whether tumor cell growth was impaired or reduced due to apoptosis, the expression of Annexin V/propidium iodide (PI) was evaluated using the Annexin V-FITC Apoptosis Detection kit (BD Pharmingen, Heidelberg, Germany). Tumor cells were washed twice with PBS, and then incubated with 5 μl of Annexin V-FITC and 5 μl of PI in the dark for 15 min at RT. Cells were analyzed by flow cytometry using FACScalibur (BD Biosciences, Heidelberg, Germany). The percentage of apoptotic (early and late), necrotic and vital cells in each quadrant was calculated using CellQuest software (BD Biosciences).

### Percentage of cells in different cell cycle phases

Cell cycle analysis was carried out on subconfluent cell cultures. Tumor cell populations were stained with PI, using a Cycle TEST PLUS DNA Reagent Kit (Becton Dickinson, Heidelberg, Germany) and then subjected to flow cytometry using FACScan (Becton Dickinson). 10,000 events were collected from each sample. Data acquisition was carried out using CellQuest software and cell cycle distribution was calculated using the ModFit software (Becton Dickinson). The number of gated cells in G1, G2/M or S-phase was expressed as %.

### Expression of cell cycle regulating proteins

Cell cycle regulating proteins were investigated by Western blot. Tumor cell lysates were applied to a 7%–15% polyacrylamide gel (depending on protein size) and electrophoresed for 90 min at 100 V. The protein was then transferred to nitrocellulose membranes (1 h, 100 V). After blocking with non-fat dry milk for 1h, the membranes were incubated overnight with monoclonal antibodies directed against the following cell cycle proteins, all from BD Biosciences, Heidelberg, Germany: Cdk1 (IgG1, clone 1, dilution 1:2,500), cdk2 (IgG2a, clone 55, dilution 1:2,500), cdk4 (IgG1, clone 97, dilution 1:250), cyclin A (IgG1, clone 25, dilution 1:250), cyclin B (IgG1, clone 18, dilution 1:1,000), cyclin D1 (IgG1, clone G124-326, dilution 1:250), Skp1 p19 (IgG1, clone 52/p19, dilution 1:5,000), p27 (IgG1, clone 57, dilution 1:500). HRP-conjugated goat anti-mouse IgG (Upstate Biotechnology, Lake Placid, NY, USA; dilution 1:5,000) served as the secondary antibody. The membranes were briefly incubated with ECL detection reagent (ECLTM, Amersham/GE Healthcare, Munich, Germany) to visualize the proteins and then analyzed by the Fusion FX7 system (Peqlab, Erlangen, Germany). ß-actin (1:1,000; Sigma, Taufenkirchen, Germany) served as internal control.

### Expression of targeted proteins

To evaluate target specificity of temsirolimus and VPA, mTOR signaling and histone acetylation were evaluated. The following monoclonal antibodies were employed to determine mTOR signaling: anti-phospho Akt (pAkt; clone 104A282, both: mouse IgG1, dilution 1:500, BD Biosciences), anti-phospho mTOR (pmTOR; IgG, Ser2448, clone D9C2, dilution 1:1,000), anti-phospho rictor (pRictor; IgG, Thr1135, D30A3, dilution 1:1,000), anti-phospho raptor (IgG, Ser792; both: MerckMillipore, dilution 1:1,000) and anti-phospho p70S6k (pp70S6k; clone 108D2, dilution 1:1,000, New England Biolabs). To investigate histone acetylation, cell lysates were marked with anti-acetylated histone H3 (rabbit IgG, clone Y28, dilution 1:500, Epitomics, USA) and anti-acetylated histone H4 (Lys8, rabbit IgG, dilution 1:500, Upstate Biotechnology, USA). HRP-conjugated goat anti-mouse or goat anti-rabbit IgG (both: dilution 1:5,000, Upstate Biotechnology, Lake Placid, NY, USA) were used as secondary antibodies. The membranes were briefly incubated with ECL detection reagent (ECL™, Amersham/GE Healthcare, Munich, Germany) to visualize the proteins and then analyzed by the Fusion FX7 system (Peqlab, Erlangen, Germany). β-actin (dilution 1:1,000, Sigma, Taufenkirchen, Germany) served as the internal control.

### Blocking studies

Since cdk2 and cyclin A revealed distinct down-regulation by VPA they might be responsible for growth inhibition induced by VPA. To determine whether cdk2 and cyclin A have an impact on growth of the used tumor cell lines, both proteins were blocked. Therefore, tumor cells (3 × 10^5^/6-well) were transfected with small interfering RNA (siRNA) directed against cdk2 (gene ID: 1017, target sequence: AGGTGGTGGCGCTTAAGAAAA) or cyclin A (gene ID: 890, target sequence: GCCAGCTGTCAGGATAATAAA) with a siRNA/transfection reagent (HiPerFect Transfection Reagent; Qiagen, Hilden, Germany) ratio of 1:6. Untreated cells and cells treated with 5 nmol control siRNA (All stars negative control siRNA; Qiagen, Hilden, Germany) served as controls. Knock-down was verified by western blot analysis. Tumor cell growth was analyzed by the MTT assay as indicated above.

### Statistics

All experiments were performed 3-6 times. Statistical significance was determined by the Wilcoxon–Mann-Whitney-*U*-test. Differences were considered statistically significant at a *p*-value less than 0.05.
